# St. Mary's Hospital Musical and Dramatic Society

**Published:** 1920-07-24

**Authors:** 


### St. Mary's Hospital Musical and Dramatic Society.

THE second entertainment of the above Society was given recently in the Library of the Medical School at 8.15 P.M. The proceedings opened with the one-act play entitled "The Twelve Pound Look," which received great applause. The *piece de resistance* was the performance given by the students in Pierrot costumes. The scene was laid in an Examination Hall, while the programme was arranged in the form of an examination paper. The object of the examiner was to discover where flies go to in the winter-time. The audience was kept in roars of laughter, and it was most refreshing to hear the candidates in the middle of their "lines" get up and sing a song to the examiners. The performers are to be congratulated on the success of their show.

